# Polypropylene Prosthesis in the Treatment of Extended Injuries and Coverage of Graft-free Donor Areas: Case Series

**DOI:** 10.1055/s-0044-1791514

**Published:** 2024-12-07

**Authors:** Raphael De Sá Vasconcelos Uchôa, Luís Filipe e Silva Lessa Ferreira, Ana Luiza Simões de Brito Uchôa

**Affiliations:** 1 Departamento de Cirurgia da Mão e Microcirurgia, Hospital Regional do Agreste, Caruaru, PE, Brasil; 2 Unidade de Trauma, Hospital Regional do Agreste, Caruaru, PE, Brasil

**Keywords:** therapeutics, wound closure techniques, wounds and injuries

## Abstract

**Objective** To evaluate the effectiveness of the use of polypropylene prostheses in the treatment of extensive limb injuries.

**Methods** There were 13 patients evaluated for the final aspects of the treatment, including the presence of epithelization and granulation, reduction of raw area, and coverage of deep structures.

**Results** A reduction greater than 40% of the raw area in all cases and complete coverage of noble structures were visualized.

**Conclusion** An effective, reproducible, low-cost alternative for treating extensive injuries has been demonstrated.

## Introduction

Injuries with integumentary loss in the limbs, associated or not with fractures, are common problems in traumatology, affecting mostly young and economically active individuals, causing them high morbidity and work absenteeism. 1 2 3 4 This context may involve factors such as prolonged hospitalization, high pharmacological cost, association with soft-tissue and bone infections, partial and precarious rehabilitation, in addition to increasing the risk of disability and death from systemic complications. 1 3 4

Moreover, these injuries are difficult to treat, taking long periods to heal, 1 3 4 or requiring complex and often inaccessible therapeutic interventions, such as plastic surgery and negative-pressure therapy. 1 5 6 7

Provisional coverage with polypropylene prosthesis has been cited as an alternative in treating these lesions and flap donor areas. The methodology gained notoriety in a recent study for demonstrating reepithelialization in fingertip lesions with loss of digital pulp; 8 however, it lacks further scientific evidence.

The present study aims to evaluate the effectiveness of polypropylene prostheses in treating extensive skin lesions on the limbs.

## Materials and Methods

The study was previously approved by the Ethics Committee of the institution (CAAE: 46264121.6.0000.5666). All participants were informed about the research and signed an informed consent form. We recruited 25 patients and interns in a tertiary hospital's Traumatology and Orthopedics Center, which used polypropylene prostheses to treat extensive injuries or cover donor flap areas. After applying the exclusion criteria, 13 patients had their results evaluated. The participants were operated on and assisted by the same group of surgeons between September and December 2021.

**Fig. 1:**
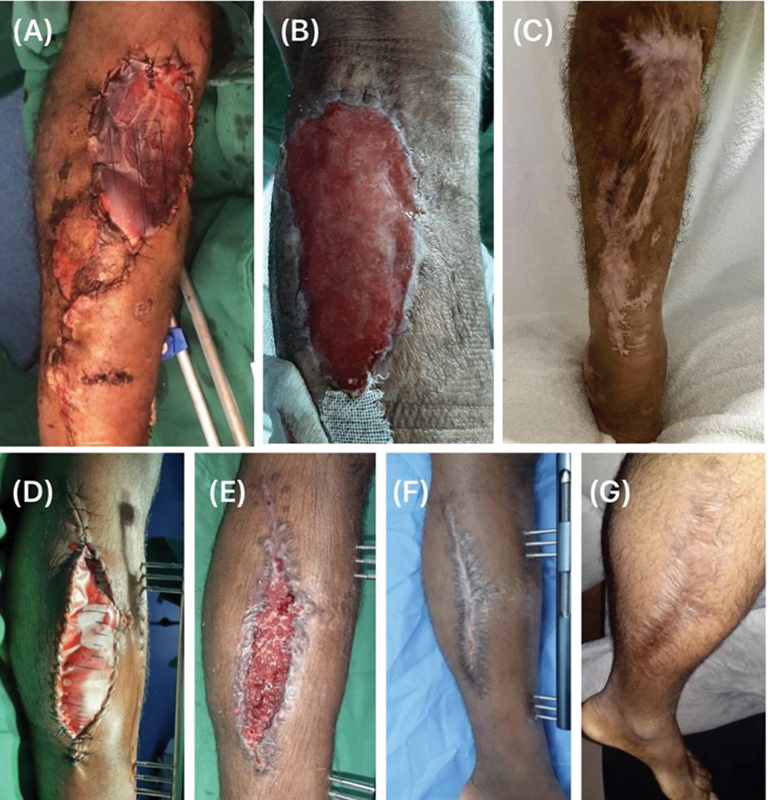
( **A** ) Patient I (48 years old) coverage of sural flap donor area with polypropylene prosthesis in 2021; ( **B** ) appearance of the wound after removal of the prosthesis at 6 weeks of evolution; ( **C** ) appearance of the resulting scar tissue in 2024; ( **D** ) patient II (22 years old) in 2021, polypropylene prosthesis on the wound on the medial side of the leg; ( **E** ) appearance of the wound 6 weeks after removal of the prosthesis, ( **F** ) the same wound 8 weeks after removal; ( **G** ) resulting scar tissue in 2024. Both reported no complaints about the scars.

The present study was designed as a long-term follow-up, in which recurrent follow-up would be carried out from the preoperative to the immediate postoperative period until removal of the prosthesis, and, finally, a last evaluation in the remote postoperative period in 2024. It was expected that after 3 years, having achieved complete maturation of the scar tissue of all participants, a reliable evaluation of data, such as structural and sensitive quality of the scar formed and related complications, could be carried out. However, after the planned period had elapsed, only 2 of the 13 patients evaluated had kept the same contact telephone number provided at the beginning. Thus, although both denied complaints and abnormalities about the scar, no definite conclusion was obtained, given the small remaining sample ( Fig. 1 ).

The inclusion criteria were patients with extensive traumatic injuries with skin loss in the upper or lower limbs, defined as areas ≥ 25 cm ^2^ (5 × 5 cm in the largest diameters) with or without exposure to noble structures (bones, nerves, tendons, and vessels). We also included individuals subjected to skin flaps, in which the donor area would need a skin graft for closure. 9

Individuals with previously infected wounds, peripheral vascular diseases, active smoking, anemia, and diabetes mellitus were excluded.

The participants were evaluated based on the protocol proposed by Figueiredo et al. 8 In this protocol, wounds with skin loss are covered with polypropylene prosthesis, which can be obtained from sterile hospital materials. The management includes maintenance of the prosthesis for an extended period, performing, from 7 days after the installation of the device, cleaning of the prosthesis and secondary coverage, with follow-up and periodic observation of the prosthesis for up to 6 weeks. 8

Debridement of the wounds and initial photographic documentation were performed. After the installation of the polypropylene prosthesis, a new photographic record was made. The photographs were obtained using a 25-megapixel camera with a resolution of 5,760 × 4,320 pixels.

The prosthesis was made on a case-by-case basis using the transparent polypropylene of the sterile bladder catheter collector to allow monitoring and documentation of healing. The prosthesis was sutured to the healthy margins of the wound through simple stitches, with a 1-cm distance between them, performed with 3–0 nylon thread, then secondary coverage. Hospital discharge was possible, in most cases, between the first and second postoperative days.

In general, the first dressing change occurred after 7 days, and from then on, daily dressing changes with appropriate cleaning of the prosthesis and the use of 70% alcohol on the stitches were advised. The participants were followed fortnightly from the first consultation. The removal of the prosthesis was scheduled between the 6 ^th^ and 7 ^th^ weeks, with the non-compliance of deep tissues to the granulation tissue as a criterion.

To evaluate the effectiveness of the treatment, we considered the reduction of the bruised area and the appearance of the resulting coverage after removal of the prosthesis. We considered a good result that which did not present residual exposure of deep structures in the wound bed. 9

**Fig. 2:**
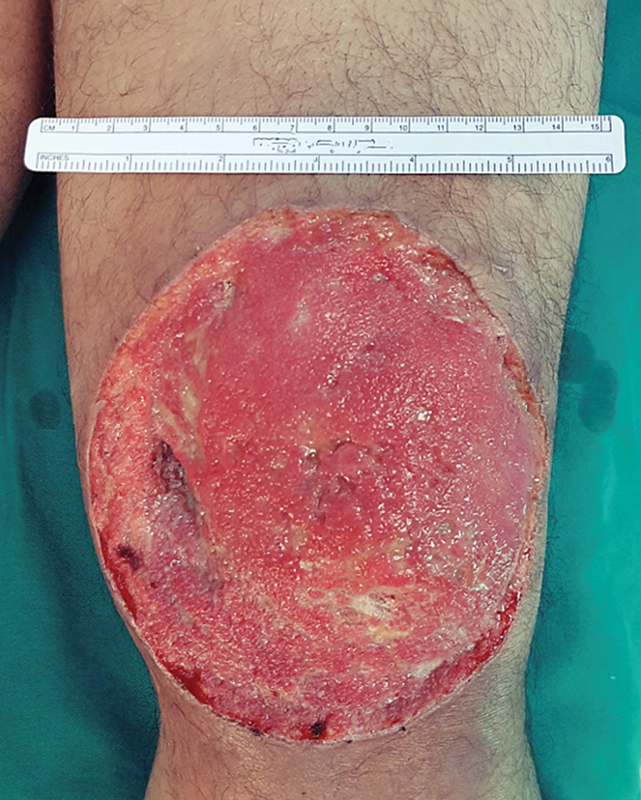
Example of a photographic record of a wound with a standard millimeter object to facilitate estimation of the wound area.

From the photographic records of each wound with a graduated standard object ( Fig. 2 ), using the software AutoCAD version 2022 (Autodesk, Inc., San Francisco, CA, USA), the comparative analysis between the initial raw area of each wound and the new measurement of the raw area after removal of the prosthesis was performed. For this purpose, the photographs were inserted in the working area of the software AutoCAD, where the edges of the wound and the standard object were demarcated, the latter with a previously known actual area. From the contours' boundary, the wound's raw area and the standard object's area were obtained in decimal numbers in the language of the software. In possession of these values and the previously known actual area of the standard object, with a simple rule of three, an estimation of the raw area of the wounds at the beginning and after removal of the prosthesis was performed in cm ^2^ . The same researcher took the measures using AutoCAD. The reduction of the measures of the raw area of the wounds of each participant was standardized in percentage for statistical analysis. The significance level considered for performing all hypothesis tests was equal to 5%.

## Results

Of the 13 participants evaluated, 11 (84.6%) were male and 2 (15.3%) were female. The median age was 31.9 years. The lesions affected the lower limbs more, with 10 patients (76.9%). The most frequent trauma mechanism was motorcycle accident, with 11 cases (84.6%), followed by 1 case of automobile accident (7.7%) and 1 case of accident with agricultural machinery (7.7%). The most affected segment was the left lower limb, with 8 cases (61.5%), followed by 2 cases of trauma to the right lower limb (15%) and 2 to the left upper limb (15%), and 1 case of injury to the right upper limb (7.7%). The epidemiological profile traced by this study follows what is reported in the literature. 9 Of the 13 patients evaluated, 4 (30%) had the initial traumatic wound treated with flaps and coverage of the donor area with polypropylene prosthesis. Of these 4, 3 were treated with flaps in the left lower limb—sural flap—and 1 (25%) in the right upper limb—Chinese flap ( Table 1 ).

**Table 1:** Descriptive analysis of qualitative and numerical variables

Wound site	Age (years)	Sex	Initial raw area(cm ^2^ )	Final raw area(cm ^2^ )	Reduction of raw area	Treatment period (weeks)
**Lateral side of left leg**	23	M	196	79	60	6
**Right forearm Chinese flap donor area**	24	M	90	17	81	8
**Back of left foot**	20	F	37.9	9.6	75	7
**Palmar side of left hand and wrist**	43	M	124	35.9	71	8
**Medial gastrocnemius flap on the proximal 1/3 of the left leg**	31	M	60.9	26	56.9	8
**Back left foot**	61	F	55	26	52	6
**Fasciotomy on the medial aspect of the left leg**	22	M	80	46	42	6
**Anterolateral side of right leg**	18	M	147	64	56	6
**Sural flap donor area on left leg**	48	M	63.5	0	100	6
**Medial surface of right leg**	18	M	59	33	43	8
**Back of the left forearm**	43	M	130	41.8	67.7	6
**Sural flap donor area on left leg**	31	M	60	25.9	56,8	6
**Sural flap donor area on left leg**	33	M	45	9.6	78.5	8

**Fig. 3:**
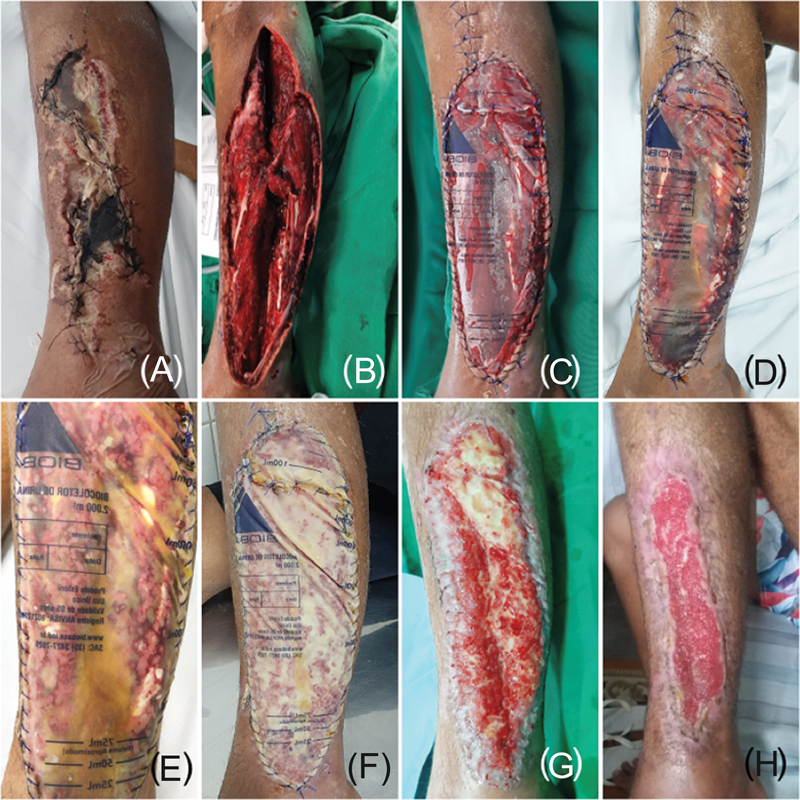
Evolutionary phases of the treatment of an extensive wound on the lateral aspect of the left leg: ( **A** ) extensive wound with devitalized tissue; ( **B** ) extensive debridement, visualizing tendinous structures, superficial peroneal nerve, and division of muscle compartments; ( **C** ) polypropylene prosthesis to cover the wound; ( **D** ) first dressing change after 7 days, noting the accumulation of serous exudate; ( **E** ) second week: it is possible to see fibrin formation and the beginning of granulation; ( **F** ) fourth week: coating of the microenvironment with thick fibrin and the occurrence of hypertrophic granulation; ( **G** ) sixth week: removal of the prosthesis and a significant reduction in the raw area, epithelialization of the wound margins, the center with hypertrophic granulation tissue, and it is not possible to see deep structures in the wound bed; ( **H** ) eighth week of follow-up, with rapid progression of epithelialization.

**Fig. 4:**
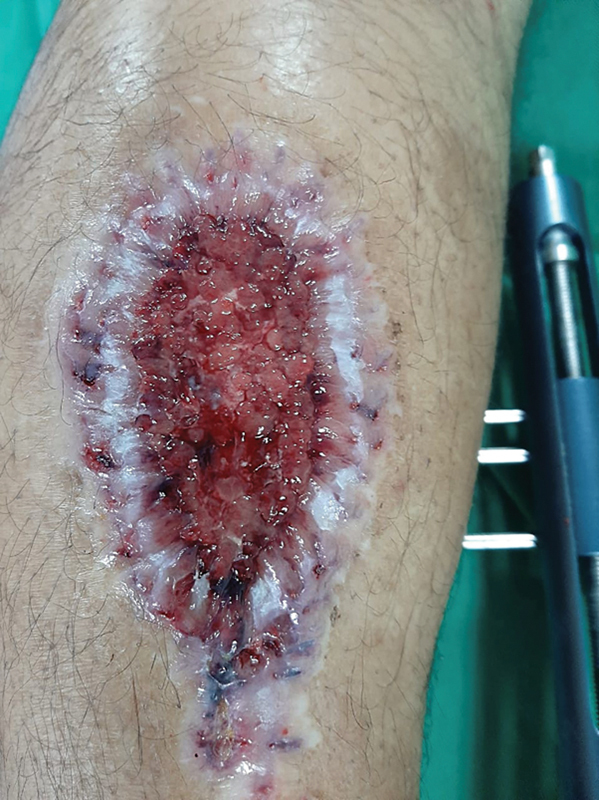
Sural flap donor area on the left leg, after removal of the polypropylene device in the 6th week, showing a reduction in the raw area, epithelialization of the edges, and a center with hypertrophic granulation tissue.

The following stages of evolution were observed among the participants, suggesting some degree of predictability of the treatment: in the first week, accumulation of serous exudate, translucent, straw yellow, and odorless. After about 2 weeks, the appearance of islands of granulation and a decrease in exudate with the formation of thick fibrin were observed, which coated the entire surface of the “microenvironment” around the 3 ^rd^ and 4 ^th^ weeks. The thick fibrin layer was gradually replaced by hypertrophic granulation tissue between the 3 ^rd^ and 4 ^th^ week. This granulation tissue fills, to a large extent, the raw area of the lesion, with less expressiveness in the initially deeper wounds or in those in which the prosthesis was in direct contact with the bone surface. After about 5 to 6 weeks, it is possible to verify the presence of reepithelialization at the wound margins, with variable thickness, under the transparent prosthesis in coexistence with granulation tissue in the central area ( Fig. 3 ). During these stages, patients presented with no clinical complaints and no systemic or local signs of infection or exuberant inflammation. The prosthesis was removed in most cases between the 6 ^th^ and 7 ^th^ week. In all cases, a reduction greater than 40% of the raw area was visualized, with epithelization of the edges of the lesion and center presenting residual granulation tissue, with measurements varying from case to case ( Fig. 4 ). In the follow-up, the rapid epithelization of the residual granulation tissue is noticeable, resulting in scar tissue of color and morphology similar to the adjacent healthy skin, painless, and with preserved sensitivity.

**Fig. 5:**
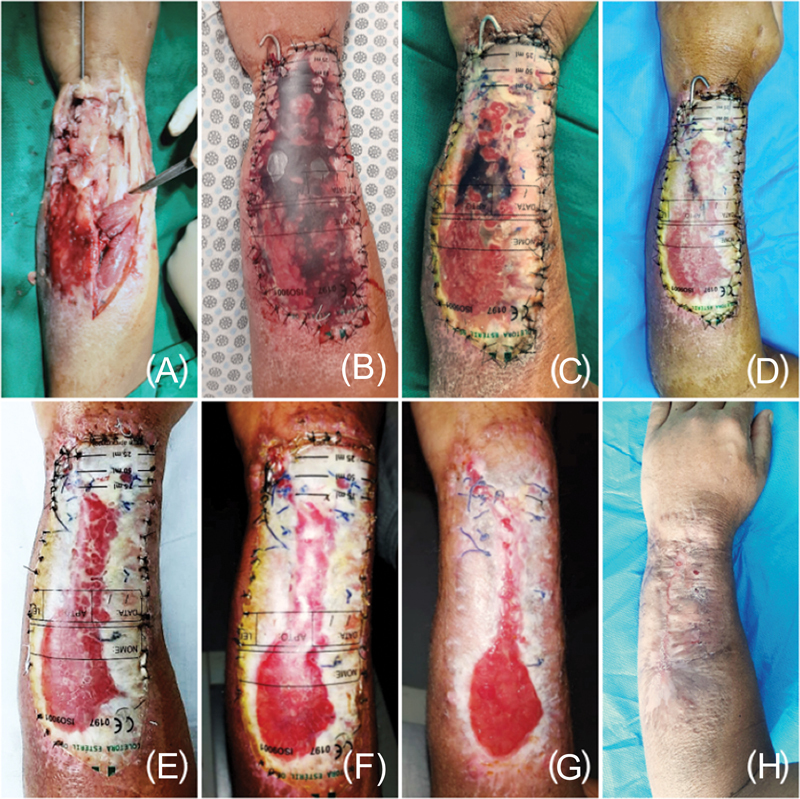
Treatment of extensive wound on the back of the left forearm: ( **A** ) initial raw area with exposure of deep structures; ( **B** ) first week; ( **C** ) second week; ( **D** ) third week; ( **E** ) fourth week; ( **F** ) sixth week: loosening of the suture anchoring the prosthesis due to scar retraction and epithelialization of the edges; ( **G** ) sixth week: removal of the device, significant reduction of the raw area, with no deep structures visible; ( **H** ) tenth week of follow-up: wound completely healed.

The healing process seemed to occur more efficiently when the prosthesis was used on the upper limbs in comparison with the lower limbs ( Fig. 5 ). This aspect was not statistically significant due to the small number of cases with upper extremity injuries.

After the removal of the prosthesis, invariably, no deep tissues were observed in the wound bed, proving the effectiveness of the prosthesis in protecting noble tissues and reducing the morbidity of traumatic injuries. However, in deep wounds, the bed filling occurred more slowly, suggesting the need for a longer prosthesis stay or indication of flaps, corroborating what was previously reported in the literature. 5 6

The average initial bruised area of the wounds was 88 cm ^**2**^ (37.9–196 cm ^**2**^ ), while the average final raw area was 31.8 cm ^**2**^ (0–79 cm ^**2**^ ). The average percentage of reduction of raw area was 64.6%. The average length of stay of the prosthesis was 6.8 weeks, ranging from 6 to 8 weeks. For statistical inference of the effectiveness of the protocol proposed by Figueiredo et al. 8 when implemented in extensive limb injuries, the portion of the sample that remained with the prosthesis for 6 weeks was analyzed. When performing the t-test for comparison of means, there was sufficient evidence to affirm that the polypropylene prosthesis reduced the raw area by more than 40% in 6 weeks, with a *p* -value equal to 0.019 and a confidence level of 95%.

There were no cases of infection during treatment with the polypropylene prosthesis, highlighting the role of prior wound care, especially when the injury is traumatic. In two cases, the formation of hypertrophic scar tissue occurred painlessly and with preserved sensitivity. In these two cases, the patients evolved with hypertrophic scarring of other adjacent raw areas not treated with the prosthesis, such as the flap pedicle region, which may suggest that this complication was not inherent to the method.

## Discussion

The technique analyzed demonstrated the advantage of not requiring a donor area for coverage, reducing morbidity associated with traumatic injuries, hospital admission, total pharmacological cost, and materials for dressings, besides the rate of infection, amputations, and other complications, as well as speeding up the return to work activities. 8

In addition, coverage with polypropylene prosthesis is an easy method that surgeons can use at the beginning of the learning curve to generate satisfactory results.

The device provides the accumulation of exudate rich in growth factors, cytokines, cellularity, proteins, and other components that create a moist microenvironment prone to faster tissue repair, restoring the anatomy of the affected region. 2 8 10 Such components, present in the humid microenvironment, are known to be essential for the scar repair process. 2 The body reacts to the device's presence with the acceleration of the healing phases in a coordinated manner, significantly impacting the healing quality. 8

Another characteristic of the humid microenvironment is the accumulation of therapeutic agents, such as antibiotics in concentrations similar to plasma through accumulated exudate, which, added to the stratum corneum formed on the wound surface inside the microenvironment, allow no or little bacterial growth. In the latter case, we are referring to non-invasive germs. 10

The main complications described are infectious processes, which have a low incidence rate when adequate debridement of devitalized tissues and decolonization of the wound with antibiotic therapy is performed before the installation of the prosthesis. 6 8

## Conclusion

The polypropylene prosthesis, used as a temporary cover for the treatment of extensive lesions in the limbs and donor areas of flaps, proved to be an effective, affordable, safe, and easy-to-perform technique. Given the encouraging results, this technique can be an alternative in services without the availability of resources such as negative-pressure dressing and lack of trained staff for reconstruction with flaps and grafts. This method can also demonstrate greater scope and versatility, requiring analysis of larger samples.
