# Supplementary material for: Polypropylene Prosthesis in the Treatment of Extended Injuries and Coverage of Graft-free Donor Areas: Case Series
Source: Rev Bras Ortop (Sao Paulo). 2024 Dec 7;59(5):e779–85. doi: 10.1055/s-0044-1791514 (PMC11624926; doi:10.1055/s-0044-1791514)
Supplement: Related texts

# Research article: Prótese de polipropileno no tratamento de lesões extensas e cobertura de áreas doadoras sem enxerto: Série de casos

**Keywords:** ferimentos e lesões, técnicas de fechamento de ferimentos, terapêutica

## Abstract

**Objetivo** Avaliar a eficácia do uso das próteses de polipropileno no tratamento de ferimentos extensos nos membros.

**Métodos** Foram avaliados 13 pacientes quanto aos aspectos finais do tratamento, incluindo presença de epitelização e granulação, redução de área cruenta e cobertura de estruturas profundas.

**Resultados** Uma redução maior que 40% da área cruenta e cobertura completa de estruturas nobres foram visualizadas.

**Conclusão** Evidenciou-se uma alternativa eficaz, reprodutível e de baixo custo no tratamento de lesões extensas.

## Introdução

Ferimentos com perda tegumentar nos membros, associados ou não a fraturas, são problemas corriqueiros na traumatologia, acometendo majoritariamente indivíduos jovens e economicamente ativos, causando-lhes alta morbidade e absenteísmo laboral. 1 2 3 4 Tal contexto pode envolver fatores como internamento hospitalar prolongado, alto custo farmacológico, associação com infecções de partes moles e ósseas, reabilitações parciais e precárias, além de aumentar o risco de invalidez e morte por complicações sistêmicas. 1 3 4

Outrossim, tais ferimentos são de difícil tratamento, levando amplos períodos para cicatrização 1 3 4 ou requerendo intervenções terapêuticas complexas e, muitas vezes, inacessíveis, como cirurgia plástica e terapia com pressão negativa. 1 5 6 7

Neste sentido, a cobertura provisória com prótese de polipropileno tem sido citada como alternativa no tratamento destas lesões e de áreas doadoras de retalhos. O método ganhou notoriedade em estudo recente por demonstrar reepitelização em lesões de ponta de dedo com perda da polpa digital; 8 no entanto, essa alternativa carece de maiores evidências científicas.

O objetivo deste estudo é avaliar a eficácia do uso das próteses de polipropileno no tratamento de lesões cutâneas extensas nos membros.

## Materiais e Métodos

O estudo foi previamente aprovado pelo Comitê de Ética da instituição (CAAE: 46264121.6.0000.5666). Todos os participantes foram informados sobre a pesquisa e assinaram um termo de consentimento livre e esclarecido. Foram recrutados 25 pacientes, internos em um Centro de Traumatologia e Ortopedia de um hospital terciário, que utilizaram prótese de polipropileno no tratamento de ferimentos extensos ou na cobertura de áreas doadoras de retalhos. Após a aplicação dos critérios de exclusão, 13 pacientes tiveram seus resultados avaliados. Os participantes foram operados e assistidos pelo mesmo grupo de cirurgiões, no período entre setembro e dezembro de 2021.

Este estudo foi idealizado como um seguimento de longo prazo, no qual seria realizado o acompanhamento recorrente do pré- ao pós-operatório imediato, até a retirada da prótese, e, por fim, uma última avaliação no pós-operatório remoto, em 2024. Esperava-se que após o período de 3 anos, tendo alcançado a maturação completa do tecido cicatricial de todos os participantes, poderia ser realizada uma avaliação fidedigna de dados como qualidade estrutural e sensitiva da cicatriz formada e complicações relacionadas. No entanto, decorrido o período planejado, apenas 2 dos 13 pacientes avaliados mantiveram o mesmo telefone de contato fornecido no início. Deste modo, embora ambos tenham negado queixas e anormalidades sobre a cicatriz, nenhuma conclusão categórica foi obtida neste sentido, visto a pequena amostra remanescente ( Fig. 1 ).

**Fig. 1:** ( **A** ) Paciente I (48 anos), cobertura de área doadora de retalho sural com prótese de polipropileno em 2021, ( **B** ) aspecto do ferimento após a retirada da prótese com 6 semanas de evolução ( **C** ) aspecto do tecido cicatricial resultante em 2024; ( **D** ) paciente II (22 anos) em 2021, prótese de polipropileno sobre ferimento na face medial da perna, ( **E** ) aspecto do ferimento com 6 semanas de evolução após a retirada da prótese, ( **F** ) o mesmo ferimento com 8 semanas de evolução, ( **G** ) tecido cicatricial resultante em 2024. Ambos não relataram queixas a respeito das cicatrizes.

Os critérios de inclusão foram portadores de ferimentos extensos traumáticos com perda cutânea nos membros superiores ou inferiores, definidos como áreas ≥ 25 cm ^2^ (5 × 5 cm nos maiores diâmetros) com ou sem exposição de estruturas nobres (ossos, nervos, tendões e vasos). Também foram incluídos indivíduos submetidos a retalhos cutâneos, nos quais a área doadora necessitaria de enxerto de pele para o fechamento. 9

Foram excluídos indivíduos com ferimentos previamente infectados, vasculopatias periféricas, tabagismo ativo, anemias e diabetes mellitus.

Os participantes foram avaliados com base no protocolo proposto por Figueiredo et al. 8 Nesse protocolo, realiza-se a cobertura de ferimentos com perda cutânea com prótese de polipropileno, a qual pode ser obtida a partir de materiais hospitalares estéreis. O manejo inclui a manutenção da prótese por período prolongado, realizando, a partir de 7 dias da instalação do dispositivo, limpeza da prótese e cobertura secundária, com acompanhamento e observação periódica da prótese até 6 semanas. 8

Foi realizado o desbridamento dos ferimentos e documentação fotográfica inicial. Após a instalação da prótese de polipropileno, foi feito um novo registro fotográfico. Para obtenção das fotografias, utilizou-se uma câmera fotográfica de 25 megapixels com resolução de 5.760 × 4.320 pixels.

A prótese foi confeccionada caso a caso utilizando o polipropileno transparente do coletor estéril de sonda vesical para permitir o acompanhamento e documentação da cicatrização. A prótese foi suturada às margens sadias do ferimento através de pontos simples, com distância de 1cm entre eles, realizados com fio de nylon 3-0, em seguida cobertura secundária. A alta hospitalar foi possível, na maioria dos casos, entre o 1° e 2° dia de pós-operatório.

Em geral, a primeira troca de curativo ocorreu após 7 dias, sendo orientado, a partir de então, trocas diárias do curativo com limpeza apropriada da prótese e uso de álcool 70% nos pontos de sutura. Os participantes foram acompanhados quinzenalmente a partir da primeira consulta. A remoção da prótese foi programada entre a 6ª e 7ª semanas, tendo como critério a inobservância de tecidos profundos ao tecido de granulação.

Para avaliação da eficácia do tratamento, considerou-se a redução da área cruenta e o aspecto da cobertura resultante após a retirada da prótese, considerando resultado bom o que não apresentou exposição residual de estruturas profundas no leito da ferida. 9

A partir dos registros fotográficos de cada ferimento com um objeto padrão milimetrado ( Fig. 2 ), utilizando o *software* AutoCAD versão 2022 (Autodesk, Inc., San Francisco, CA, EUA), foi realizada a análise comparativa entre a área cruenta inicial de cada ferimento e a nova medida de área cruenta após a retirada da prótese. Para tanto, as fotografias foram inseridas na área de trabalho do *software* AutoCAD, na qual realizou-se a demarcação dos bordos da ferida e do objeto padrão, este último com área real previamente conhecida. A partir da demarcação dos contornos, foram obtidas a área cruenta da ferida e a área do objeto padrão em números decimais na linguagem do *software* . Em posse desses valores e da área real previamente conhecida do objeto padrão, com regra de três simples, foi realizada a estimativa da área cruenta das feridas no início e após a retirada da prótese em cm ^2^ . As medidas no AutoCAD foram feitas pelo mesmo pesquisador. A redução das medidas de área cruenta das feridas de cada participante foi padronizada em percentual para análise estatística. O nível de significância considerado para realizar todos os testes de hipóteses foi igual a 5%.

**Fig. 2:** Exemplo de registro fotográfico de ferimento com objeto padrão milimetrado para facilitar estimativa da área cruenta

## Resultados

Dos 13 participantes avaliados 11 (84,6%) eram do sexo masculino e 2 (15,3%) do sexo feminino. A média de idade foi de 31,9 anos. As lesões acometeram mais os membros inferiores, com um total de 10 pacientes (76,9%). O mecanismo de trauma mais frequente foi acidente motociclístico, com 11 casos (84,6%), seguido de 1 caso de acidente automobilístico (7,7%) e um caso de acidente com máquina agrícola (7,7%). O segmento mais acometido foi o membro inferior esquerdo, com 8 casos (61,5%), seguido por 2 casos de trauma no membro inferior direito (15%) e 2 no membro superior esquerdo (15%), e 1 caso de ferimento no membro superior direito (7,7%). O perfil epidemiológico traçado por este estudo está de acordo com o que é relatado na literatura. 9 Dos 13 pacientes avaliados, 4 (30%) tiveram o ferimento traumático inicial tratado com retalhos e cobertura da área doadora com prótese de polipropileno. Destes 4, 3 foram de retalho no membro inferior esquerdo (retalho sural) e 1 (25%) no membro superior direito (retalho chinês) ( Tabela 1 ).

**Tabela 1:** Análise descritiva de variáveis qualitativas e numéricas

Local do ferimento	Idade (anos)	Sexo	Área cruenta inicial (cm 2 )	Área cruenta final (cm 2 )	Redução da área cruenta (%)	Tempo de tratamento (semanas)
**Face lateral da perna esquerda**	23	M	196	79	60	6
**Área doadora de retalho chinês no antebraço direito**	24	M	90	17	81	8
**Dorso do pé esquerdo**	20	F	37,9	9,6	75	7
**Face palmar da mão e punho esquerdo**	43	M	124	35,9	71	8
**Retalho de gastrocnêmio medial no 1/3 proximal da perna esquerda**	31	M	60,9	26	56,9	8
**Dorso do pé esquerdo**	61	F	55	26	52	6
**Fasciotomia na fae medial da perna esquerda**	22	M	80	46	42	6
**Face anterolateral da perna direita**	18	M	147	64	56	6
**Área doadora de retalho sural na perna esquerda**	48	M	63,5	0	100	6
**Face medial da perna direita**	18	M	59	33	43	8
**Dorso do antebraço esquerdo**	43	M	130	41,8	67,7	6
**Área doadora de retalho sural na perna esquerda**	31	M	60	25,9	56,8	6
**Área doadora de retalho sural na perna esquerda**	33	M	45	9,6	78,5	8

Observou-se as seguintes etapas de evolução entre os participantes, sugerindo algum grau de previsibilidade do tratamento: na primeira semana, acúmulo de exsudato seroso, translúcido, amarelo palha e inodoro. Após cerca de 2 semanas, observou-se o surgimento de ilhas de granulação, diminuição do exsudato, com formação de fibrina espessa, a qual revestiu toda a superfície do “microambiente” por volta da 3ª e 4ª semanas. Gradativamente ocorreu a substituição da camada de fibrina espessa por tecido de granulação hipertrófica, com início entre a 3ª e a 4ª semana. Este tecido de granulação preenche, em grande parte, a área cruenta da lesão, com menos expressividade nos ferimentos inicialmente mais profundos, ou naqueles em que a prótese esteve em contato direto com superfície óssea. Após cerca de 5 a 6 semanas, é possível verificar presença de reepitelização nas margens do ferimento, com espessura variável, sob a prótese transparente, em coexistência com tecido de granulação na área central ( Fig. 3 ). Durante essas etapas, os pacientes não apresentaram queixas clínicas nem sinais sistêmicos ou locais de infecção ou inflamação exuberante. A retirada da prótese foi realizada, na maioria dos casos, entre a 6ª e 7ª semana, sendo visualizado em todos os casos redução maior que 40% da área cruenta, com epitelização dos bordos da lesão e centro apresentando tecido de granulação residual, com medidas variando caso a caso ( Fig. 4 ). No seguimento, é notória a rapidez na epitelização do tecido de granulação residual, resultando em um tecido cicatricial de coloração e morfologia semelhante à pele saudável adjacente, indolor e com sensibilidade preservada.

**Fig. 3:** Fases evolutivas do tratamento de ferimento extenso na face lateral da perna esquerda: (a) ferimento extenso com tecido desvitalizado; (b) desbridamento extenso, visualizando-se estruturas tendíneas, nervo fibular superficial e divisão dos compartimentos musculares; (c) aposição de prótese de polipropileno para cobertura do ferimento; (d) primeira troca de curativo após 7 dias, sendo notado o acúmulo de exsudato seroso; (e) segunda semana: é possível visualizar formação de fibrina e início da granulação; (f) quarta semana: revestimento do microambiente com fibrina espessa e ocorrência de granulação hipertrófica; (g) sexta semana: retirada da prótese e visualizado redução expressiva da área cruenta, epitelização das margens da ferida, centro com tecido de granulação hipertrófica, não sendo possível visualizar estruturas profundas no leito da ferida; (h) oitava semana de seguimento, com progressão rápida da epitelização.

**Fig. 4:** Área doadora de retalho sural na perna esquerda, após retirada do dispositivo de polipropileno, na sexta semana, visualizando-se redução da área cruenta, epitelização dos bordos e centro com tecido de granulação hipertrófico.

O processo de cicatrização pareceu ocorrer de forma mais eficiente quando a prótese foi utilizada nos membros superiores em relação aos inferiores ( Fig. 5 ). Esse aspecto não teve significância estatística devido ao pequeno número de casos com lesões nas extremidades superiores.

**Fig. 5:** Tratamento de ferimento extenso no dorso do antebraço esquerdo: (a) área cruenta inicial com exposição de estruturas profundas; (b) primeira semana; (c) segunda semana; (d) terceira semana; (e) quarta semana; (f) sexta semana: observa-se soltura da sutura de ancoragem da prótese devido à retração cicatricial e epitelização dos bordos; (g) sexta semana: remoção do dispositivo, observa-se redução significativa da área cruenta, não sendo possível visualizar estruturas profundas; (h) décima semana de seguimento: ferimento completamente cicatrizado.

Após a remoção da prótese, invariavelmente, não foram observados tecidos profundos no leito das feridas, comprovando a eficácia da prótese na proteção de tecidos nobres e redução da morbidade de ferimentos traumáticos. No entanto, nos ferimentos de grande profundidade, o preenchimento do leito se deu de forma mais lenta, sugerindo necessidade de permanência da prótese por maior tempo ou indicação de retalhos, corroborando o que foi previamente relatado na literatura. 5 6

A média da área cruenta inicial dos ferimentos foi de 88 cm ^**2**^ (37,9–196 cm ^**2**^ ) **,** enquanto a média da área cruenta final foi de 31,8 cm ^**2**^ (0–79 cm ^**2**^ ). O percentual médio de redução de área cruenta foi de 64,6%. Quanto ao período de permanência médio da prótese, foi de 6,8 semanas, variando de 6 a 8 semanas. Para inferência estatística da eficácia do protocolo proposto por Figueiredo et al. 8 quando implementado em ferimentos extensos nos membros, analisou-se a parcela da amostra que permaneceu com prótese por 6 semanas. Ao realizar o teste t para comparação de médias, houve evidências suficientes para afirmar que o uso da prótese de polipropileno proporcionou redução da área cruenta maior que 40% em 6 semanas, com valor de *p* igual a 0,019 e nível de confiança de 95%.

Não foram verificados casos de infecção no decorrer do tratamento com a prótese de polipropileno, ressaltando o papel dos cuidados prévios com o ferimento, sobretudo quando a lesão for de natureza traumática. Em dois casos ocorreu a formação de tecido cicatricial hipertrófico, indolor e com sensibilidade preservada. Nesses dois casos, os pacientes evoluíram com cicatrização hipertrófica de outras áreas cruentas adjacentes que não foram tratadas com o dispositivo, a exemplo da região do pedículo do retalho, o que pode sugerir que essa complicação não foi inerente ao método.

## Discussão

A técnica analisada demonstrou como vantagem não necessitar de área doadora para cobertura, reduzindo a morbidade associada a ferimentos traumáticos, bem como redução do internamento hospitalar, custo total farmacológico e com materiais para curativos, índice de infecção, amputações e outras complicações, bem como celeridade no retorno às atividades laborais. 8

Além disso, a utilização de cobertura com prótese de polipropileno é um método de fácil realização, podendo ser utilizado por cirurgiões no início de curva de aprendizado, gerando resultados satisfatórios.

A utilização do dispositivo proporciona o acúmulo de exsudato rico em fatores de crescimento, citocinas, celularidade, proteínas e outros componentes que criam um microambiente úmido propenso à reparação tecidual mais rápida, restaurando a anatomia da região acometida. 2 8 10 Tais componentes presentes no microambiente úmido, são sabidamente essenciais para o processo de reparação cicatricial. 2 O organismo reage à presença do dispositivo com aceleração das fases de cicatrização de forma coordenada e com impacto expressivo na qualidade da cicatrização. 8

Outra característica do microambiente úmido é o acúmulo de agentes terapêuticos, como antibióticos em concentrações semelhantes à plasmática através do exsudato acumulado, o que, somado ao estrato córneo formado na superfície do ferimento, no interior do microambiente, permite nenhum ou pouco crescimento bacteriano. Neste último caso, estamos nos referindo a germes não invasivos. 10

As principais complicações descritas são processos infecciosos, os quais têm baixa taxa de incidência quando é realizado adequado desbridamento de tecidos desvitalizados e descolonização do ferimento com antibioticoterapia antes da instalação da prótese. 6 8

## Conclusão

A prótese de polipropileno como cobertura provisória do tratamento de lesões extensas nos membros e áreas doadoras de retalhos demonstrou ser uma técnica eficaz, de custo acessível, segura e de fácil execução. Diante dos resultados encorajadores, esta técnica pode ser uma alternativa em serviços sem disponibilidade de recursos como curativo por pressão negativa e ausência de equipe capacitada para reconstrução com retalhos e enxertos. Esse método pode ainda demonstrar maior abrangência e versatilidade, necessitando análise de maiores amostras.
